# Optimization of Photothermal Catalytic Reaction of Ethyl Acetate and NO Catalyzed by Biochar-Supported MnO_x_-TiO_2_ Catalysts

**DOI:** 10.3390/toxics12070478

**Published:** 2024-06-30

**Authors:** Hongqiang Wang, Huan Zhang, Luye Wang, Shengpeng Mo, Xiaobin Zhou, Yinian Zhu, Zongqiang Zhu, Yinming Fan

**Affiliations:** 1 School of Environmental Science and Engineering, Guilin University of Technology, Guilin 541000, China; sjhjgc@163.com (H.W.); zh15357211876@163.com (H.Z.); wly15877199434@163.com (L.W.); moshengpeng14@mails.ucas.ac.cn (S.M.); zhouxiaobin@glut.edu.cn (X.Z.); zhuyinian@glut.edu.cn (Y.Z.); zhuzongqiang@glut.edu.cn (Z.Z.); 2 The Guangxi Key Laboratory of Theory and Technology for Environmental Pollution Control, Guilin University of Technology, Guilin 541000, China; 3 Guangxi Collaborative Innovation Center for Water Pollution Control and Water Safety in Karst Areas, Guilin 541004, China

**Keywords:** biochar, load, photothermocatalysis, EA, NO

## Abstract

The substitution of ethyl acetate for ammonia in NH_3_-SCR provides a novel strategy for the simultaneous removal of VOCs and NO. In this study, three distinct types of biochar were fabricated through pyrolysis at 700 °C. MnO_x_ and TiO_2_ were sequentially loaded onto these biochar substrates via a hydrothermal process, yielding a family of biochar-based catalysts with optimized dosages. Upon exposure to xenon lamp irradiation at 240 °C, the biochar catalyst designated as 700-12-3GN, derived from Ginkgo shells, demonstrated the highest catalytic activity when contrasted with its counterparts prepared from moso bamboo and loofah. The conversion efficiencies for NO and ethyl acetate (EA) peaked at 73.66% and 62.09%, respectively, at a catalyst loading of 300 mg. The characterization results indicate that the 700-12-3GN catalyst exhibits superior activity, which can be attributed to the higher concentration of Mn^4+^ and Ti^4+^ species, along with its superior redox properties and suitable elemental distribution. Notably, the 700-12-3GN catalyst has the smallest specific surface area but the largest pore volume and average BJH pore size, indicating that the specific surface area is not the predominant factor affecting catalyst performance. Instead, pore volume and average BJH pore diameter appear to be the more influential parameters. This research provides a reference and prospect for the resource utilization of biochar and the development of photothermal co-catalytic ethyl acetate and NO at low cost.

## 1. Introduction

Volatile organic compounds (VOCs) and nitrogen oxides (NO_x_s) are unorganized emissions resulting from the catalytic cracking of fossil crude oil during industrial production [1,2]. VOCs and NOxs not only have significant bioaccumulation potential and toxicity but also serve as important precursors to secondary atmospheric pollution, such as photochemical smog, acid rain, PM 2.5, and ozone depletion [3,4]. In light of this, many researchers have started to investigate methods for removing VOCs and NOxs, such as photocatalysis, thermal catalysis, and photothermal catalysis [5,6,7,8]. The most common method for removing NO_x_ is catalytic reduction technology (NH_3_-SCR), which uses NH_3_ as a reducing agent, while for removing VOCs, catalytic oxidation technology (RCO) is employed, using oxygen as an oxidizing agent. However, these methods do not achieve a high removal rate for both NO_x_ and VOCs. Noting that NO is oxidizing and VOCs are reducing in nature, a photothermal synergistic catalytic approach can be used to compensate for the low efficiency of photocatalysis and high energy consumption of thermal catalysis, thereby removing VOCs along with NO_x_ [5,9,10,11]. The use of VOCs instead of NH_3_ effectively prevents the leakage of NH_3_, thus avoiding the secondary pollution issues that it can cause [12].

Manganese oxides (MnO_x_) are widely used as catalysts for the catalytic degradation of nitrogen oxides and VOCs due to their great variety, availability of valence states, high stability, and high oxygen storage capacity [6,13,14]. Wang et al. [15] prepared cerium–manganese solid solutions using the sol–gel method to prepare cerium–manganese solid solution catalysts. Among these, Ce_0.25_Mn_0.75_O_x_ demonstrated excellent photothermal catalytic activity for the degradation of gaseous acetone under visible–infrared irradiation (150 mW/cm^2^), with a reaction rate constant 11 times higher than that of MnO_x_. Wang et al. [16] developed a new catalyst for the degradation of gaseous acetone using 2D-structured graphene oxide (GO), manganese oxide (MnO_x_), and polymerized carbon nitride (CN) nanosheets as building blocks. The superior photothermal effect of GO could rapidly increase the surface temperature of the catalyst under light, thereby driving the thermal catalysis of manganese oxide. In addition, the temperature increases accelerated charge transfer and surface reactions in the photocatalyst. As a result, the thin-film catalyst exhibited improved photothermal and photocatalytic synergistic effects in decomposing formaldehyde (HCHO), and its catalytic activity was much higher than that of individual thermal, photothermal, and photocatalysts. TiO_2_, as a star of photocatalysis, has long been popular and well-received due to its advantages, including chemical stability, strong redox properties, nontoxicity, and low cost [17,18]. Especially under UV irradiation, TiO_2_ can exhibit “superhydrophobic” properties on its surface, which prevents water molecule agglomeration and enables the oxidization of H_2_O to OH- [19,20,21]. The development of biochar (BC) has a long history in human civilization, where plant residues have long been subjected to high-temperature thermal cracking to obtain biochar. Biochar has also become a focal point in photocatalyst research due to its diverse sources, ease of preparation, and stable chemical properties. Zhou et al. [22] prepared a composite catalyst (CuBi_2_O_4_/BiOBr/BC) by incorporating CuBi_2_O_4_/BiOBr into cotton stalk biochar and investigated the photocatalytic degradation of benzene using this catalyst under visible light irradiation. It was found that the photocatalytic degradation performance of the CuBi_2_O_4_/BiOBr/BC catalyst surpassed that of the CuBi_2_O_4_, CuBi_2_O_4_/BC, and CuBi_2_O_4_/BiOBr composites. Furthermore, the introduction of biochar and the formation of Z-type heterojunctions not only enhanced the light absorption and response ranges of the nanocatalysts but also facilitated the separation of the photogenerated carriers under the combined effect. Zhang et al. [23] prepared Ag/ZnO/nBC for the photocatalytic degradation of formaldehyde, and they found that the degradation efficiency of the photocatalysts with BC was higher than that of those without BC. This was attributed to the fact that BC contained abundant surface functional groups (-OH, -CO, and -CO) and had mesoporous and macroporous structures induced by its hierarchical structure, allowing for the rapid capture and dynamic diffusion of formaldehyde.

Ginkgo shells possess the advantages of well-developed pores, a hard texture, and a large specific surface area, making them good sources of carriers for material preparation [24]. Moso bamboo has a complex and multilevel microstructure, qualifying it as a good source of biochar materials [25]. Like Ginkgo shells and moso bamboo, loofah also boasts a porous microstructure and excellent mechanical strength [26]. In light of this, this paper uses ethyl acetate (EA), a typical VOC, as a reducing agent for the catalytic reduction of NO, replacing ammonia. This approach achieves the simultaneous removal of EA and NO_x_ through photothermal coupling, thereby conserving ammonia. In this paper, TiO_2_ and MnO_x_ were loaded onto three k of biochar by the hydrothermal method. The intrinsic connection between the activity of the 700-12-3GN catalyst and its redox performance was elucidated by optimizing the preparation conditions, the type of biochar, and the loading amount. Additionally, the physicochemical properties of the catalysts were analyzed using characterization techniques such as FTIR, XRD, XPS, BET, H_2_-TPR, and O_2_-TPD, thereby providing a theoretical foundation for the synergistic catalysis of NO and EA.

## 2. Experiment

### 2.1. Catalyst Preparation

Biochar was prepared from white ginkgo shell (GN), loofah (LF), and moso bamboo (MB), and catalysts loaded with manganese and titanium were prepared using these three biochars, with varying dosages of biochar of 100 mg, 300 mg, and 500 mg, respectively. The prepared catalysts were labeled 700-12-XYY. Here, 700 represents the roasting temperature of the biochar, 12 represents the hydrothermal time, X represents the amount of biochar added, and YY represents the type of biochar. For example, 700-12-5GN represents the ginkgo shell biochar obtained by roasting at 700 °C, adding 500 mg, and obtaining the supported mangano-titanium biochar catalyst by hydrothermal reaction for 12 h. The methods are described in detail in Supplementary Material.

### 2.2. Evaluation of Catalyst Activity

For the synthesized catalyst placed in a co-catalytic reaction system irradiated by a xenon light source, 500 mg of catalyst was taken and then exposed to the simulated flue gas while being irradiated by a light source (PLS-SXE300+/300+UV). The simulated flue gasses were 7 mL/min NO, 200 mL/min O_2_, 100 mL/min EA, and 400 mL/min carrier gas with a gas hourly space velocity (GHSV) of 120,000 mL/(g·h). The concentrations of the reactant and product gasses were monitored by an online GC-6600 gas chromatograph (Fanwei, China) and a low concentration automatic smoke analyzer (Junray, model ZR-3260D, Qingdao, China). The device is shown in Figure S1. Each temperature point was stabilized for 50 min and then recorded. The conversion rates of NO and EA were calculated according to the following equations, respectively:

(1) $$X_{\mathrm{NO}}=1-\frac{C_{\mathrm{NO},\mathrm{out}}}{C_{\mathrm{NO},\mathrm{in}}}\times 100\%$$

(2) $$X_{\mathrm{EA}}=1-\frac{C_{\mathrm{EA},\mathrm{out}}}{C_{\mathrm{EA},\mathrm{in}}}\times 100\%$$

The equations X_NO_, X_EA_, C_NO,out_, C_NO,in_, C_EA,out_, C_EA,in_ are the conversion rate of NO (%), the conversion rate of EA (%), the concentration of NO outlet, the concentration of NO inlet, the concentration of EA outlet, and the concentration of EA inlet, respectively.

### 2.3. Catalyst Characterization

The structural properties of the catalysts were characterized and analyzed using X-ray diffraction (XRD), specific surface area testing (BET), and scanning electron microscopy (SEM). The chemical properties of the catalysts were analyzed by X-ray photoelectron spectroscopy (XPS), H_2_temperature-programmed reduction (H_2_-TPR), O_2_temperature-programmed desorption (O_2_-TPD), and Fourier transform infrared spectroscopy (FTIR). Detailed characterization and methods are described in the Supplementary Materials.

## 3. Results and Discussion

### 3.1. Catalyst Performance

**Figure 1:**
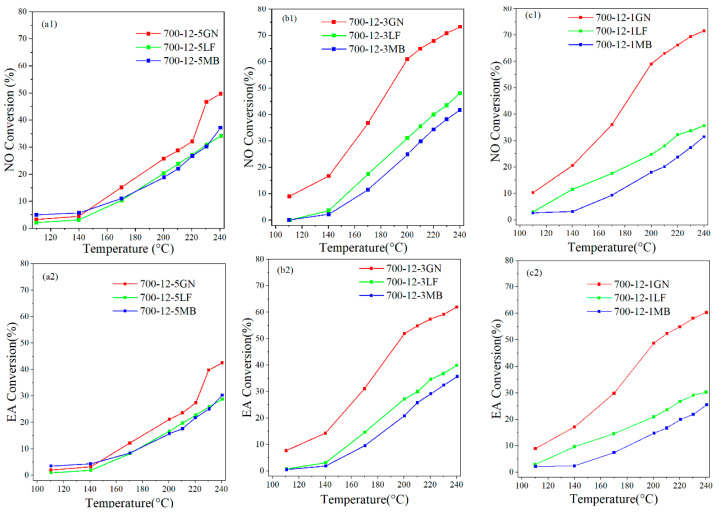
The conversion efficiencies of catalytic with temperature. (**a1**,**a2**) are the NO conversion efficiency and EA conversion efficiency for 700-12-5, respectively; (**b1**,**b2**) are the NO conversion efficiency and EA conversion efficiency for 700-12-3, respectively; (**c1**,**c2**) are the NO conversion efficiency and EA conversion efficiency for 700-12-1, respectively.

**Figure 2:**
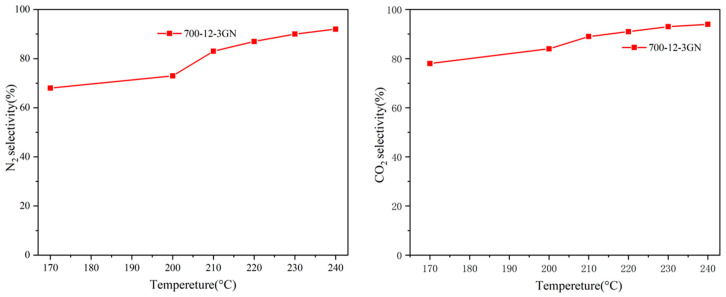
N_2_ and CO_2_ selectivity for 700-12-3GN.

The performance of the 700-12-5GN, 700-12-5LF, 700-12-5MB, 700-12-3GN, 700-12-3LF, 700-12-3MB, 700-12-1GN, 700-12-1LF, and 700-12-1MB catalysts for the degradation of NO and EA is evaluated in Figure 1. As can be seen from Figure 1, the conversion efficiency was optimal when the dosage of biochar was 300 mg. At 240 °C under xenon lamp irradiation, the conversions of NO and EA were 73.66% and 62.09% for 700-12-3GN, 48.78% and 40.29% for 700-12-3LF, and 42.35% and 35.81% for 700-12-3MB, respectively. After 700-12-3GN catalytic treatment, the concentration of ethyl acetate in the tail gas was approximately 1.32 ppm (240 °C). The catalytic performances of the three for NO and EA degradation were ranked in the following order: 700-12-3GN > 700-12-3LF > 700-12-3MB. Figure S2 shows the temperature change curve of 700-12-3GN catalyst activity. It can be found that in the case of temperature only, the activity of the 700-12-3GN catalyst is far less than that in the photothermal case. As shown in Figure 2, the N_2_ and CO_2_ selectivity of the 700-12-3GN catalyst can reach more than 80% at 210 °C and does not decrease with the increased temperature. Therefore, 700-12-3GN, 700-12-3LF, and 700-12-3MB were selected for the characterization analysis. 

### 3.2. Catalyst Structure Characterization

**Figure 3:**
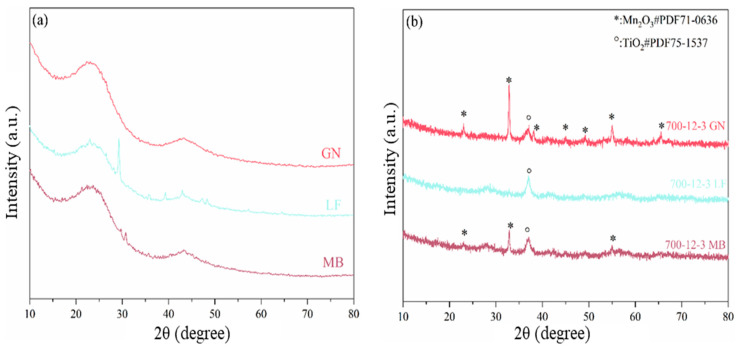
(**a**) The XRD patterns of biocha, (**b**) The XRD patterns of catalysts.

The crystalline structure of the catalyst was investigated using X-ray diffraction (XRD) and Figure 3 presents the XRD image of the catalyst. Among these, Figure 3a illustrates the XRD patterns of biochars GN, LF, and MB. Samples GN, LF, and MB exhibit characteristic peaks at 22.5°, which correspond to the characteristic peaks of cellulose [27]. As shown in Figure 3b, samples 700-12-3GN, 700-12-3LF, and 700-12-3MB share a characteristic peak at 37.5° [28], which is attributed to the (103) crystallographic plane of the anatase TiO_2_ phase(JCPDS PDF#75-1537, the diffraction peak is marked with “*”), while 32.9°, 38.2°, 45.2°, and 65.7° in sample 700-12-3GN correspond to the (103), (400), (332), and (622) crystal planes of the cubic Mn_3_O_4_ phase(JCPDS PDF#71-0636, the diffraction peak is marked with “°”), respectively [29,30]. In comparison to 700-12-3LF and 700-12-3MB, sample 700-12-3GN exhibits higher peak intensity and narrower peaks. This is also the reason for the small specific surface area of the 700-12-3GN catalyst. 

**Figure 4:**
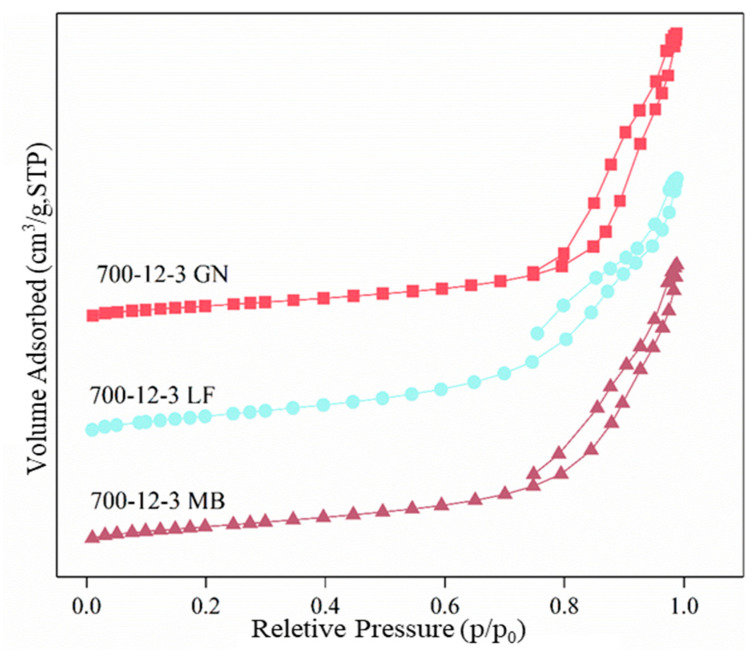
N_2_ adsorption–desorption isotherms for all catalysts.

Table 1 provides the individual BET data for the samples. The table indicates that the specific surface area of all three samples differs, which may be attributed to the varying specific surface areas of different types of biochar. Among these, 700-12-3GN has the smallest specific surface area, measuring 53.65 m^2^/g, while its pore volume and the average pore diameter according to the BJH method are the largest among the three catalysts, reaching 0.254 cm^3^/g and 17.3 nm, respectively. It is evident that the magnitude of the specific surface area is not the sole determinant of catalytic performance, while the pore volume and the average pore diameter of the BJH are likely to be influential factors in the performance of the catalysts. This may be attributed to the presence of a high number of reactive species on the 700-12-3GN catalyst. Figure 4 displays the N_2_ adsorption–desorption isotherms for the catalysts. The N_2_ adsorption–desorption isotherms of all three catalysts were classified as type IV adsorption isotherms with H3 hysteresis loops, and the presence of H3 hysteresis loops suggests an irregular pore structure and the presence of abundant ordered mesoporous structures [31]. 

**Figure 5:**
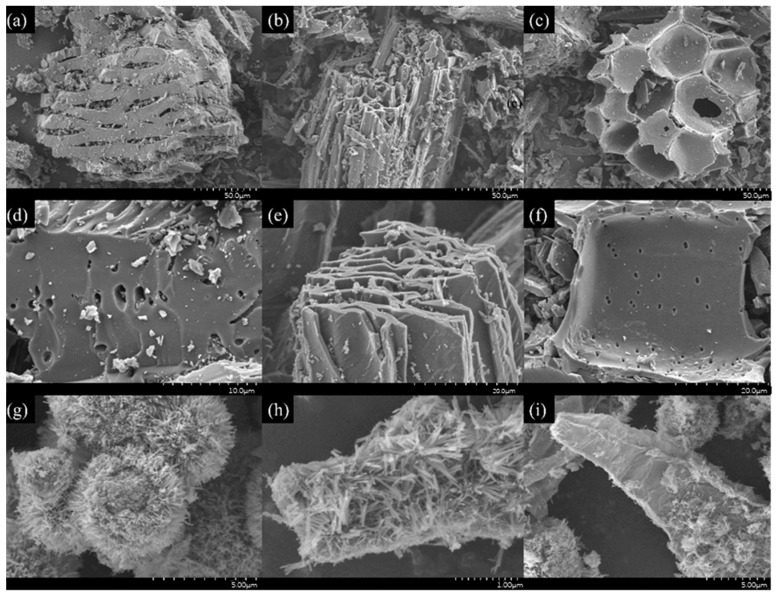
SEM images of the catalysts. (**a**,**d**): GB; (**b**,**e**): LF; (**c**,**f**): MB; (**g**): 700-12-3GN; (**h**): 700-12-3LF; (**i**): 700-12-3MB.

**Figure 6:**
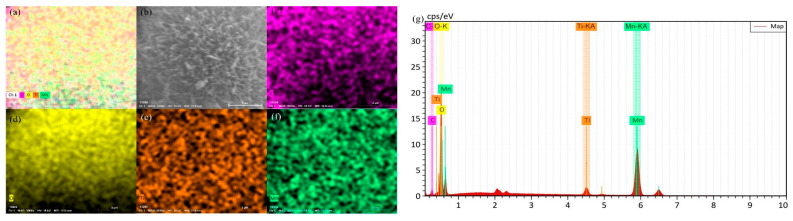
EDS energy spectra of 700-12-3 GN. (**a**): The full spectrum of each element, (**b**): the pick point map, (**c**): the distribution of C elements, (**d**): the distribution of O elements, (**e**): the distribution of Ti elements, (**f**): the distribution of Mn elements, and (**g**): the energy spectrum of each element.

**Figure 7:**
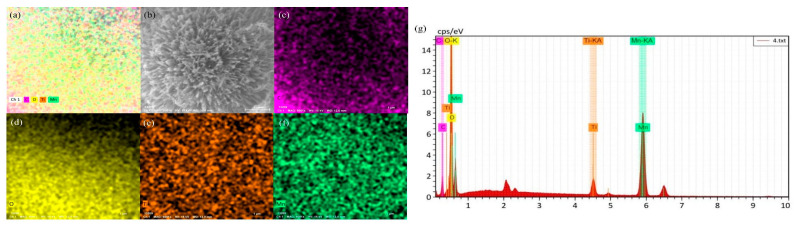
EDS energy spectra of 700-12-3 LF. (**a**): The full spectrum of each element, (**b**): the pick point map, (**c**): the Distribution of C elements, (**d**): the distribution of O elements, (**e**): the distribution of Ti elements, (**f**): the distribution of Mn elements, and (**g**): the energy spectrum of each element.

**Figure 8:**
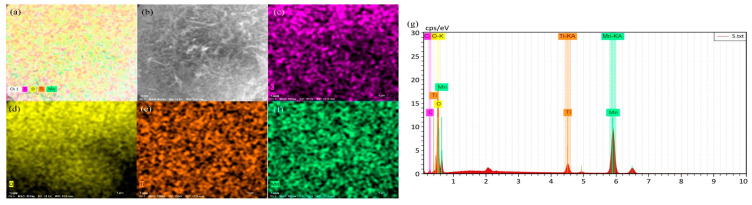
EDS energy spectra of 700-12-3 MB. (**a**): The full spectrum of each element, (**b**): the pick point map, (**c**): the distribution of C elements, (**d**): the distribution of O elements, (**e**): the distribution of Ti elements, (**f**): the distribution of Mn elements, and (**g**): the energy spectrum of each element.

The morphology of 700 °C pyrolyzed biochar GN, LF, and MB, along with three catalysts, 700-12-3GN, 700-12-3LF, and 700-12-3MB, was investigated using SEM observation, as shown in Figure 5. After 700 °C pyrolysis, the surface of the biochar becomes rough, and there are tiny particles on the surface. As can be seen in Figure 5a–c, the microstructures of the different biochars varied; after magnification, as seen in Figure 5d–f, irregularly distributed small pores were observed on the surfaces of GN, LF, and MB. The SEM images of 700-12-3GN, 700-12-3LF, and 700-12-3MB are depicted in Figure 5g–i, respectively. These images reveal that the surface of the biochar displays a morphology characteristic of rod-like spheres, suggesting that MnO_x_ and TiO_2_ have been loaded onto the biochar surface, using the biochar as a carrier. The elemental distributions and energy spectra of the individual samples are presented in Figure 6, Figure 7 and Figure 8, which confirm that the three catalysts exhibit similar elemental distributions. The EDS energy spectra of 700-12-3GN confirm the presence of the elements Ti and Mn on the biochar, with corresponding signals identified in the spectrograms. The loading of Mn-Ti species is expected to significantly enhance the activity of the catalyst.

### 3.3. Chemical Characterization of Catalysts

X-ray photoelectron spectroscopy (XPS) enables a more detailed analysis of the chemical state of the prepared catalysts. The comprehensive XPS spectrum of the sample is presented in Figure S3. The elements of Mn 2p, O 1s, Ti 2p, and C 1s are present on the surface of the sample. In comparison, 700-12-3GN exhibits the highest contents of elemental O and Mn, which are 51.4% and 22.13%, respectively. The content of elemental O in 700-12-3GN is 1.08 times higher than that in 700-12-3LF and 1.02 times higher than that in 700-12-3MB; the content of elemental Mn is 1.22 times higher than that in 700-12-3LF and 1.04 times higher than that in 700-12-3MB. The precise values of the content of each element are listed in Table 2.

**Figure 9:**
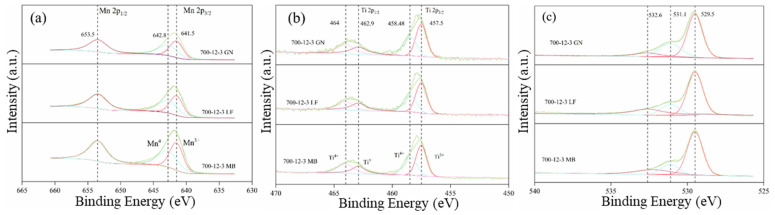
XPS fine spectra of each element, (**a**): Mn 2p; (**b**): Ti 2p; (**c**): O 1s.

The XPS fine spectrum was utilized to characterize the samples’ elemental chemical state and surface oxygen species. Figure 9 presents the XPS fine spectra of Mn 2p, Ti 2p, and O 1s. As shown in Figure 9a, the Mn 2p energy spectrum reveals a spin–orbit double state consisting of Mn 2p_1/2_ and Mn 2p_3/2_ peaks with binding energies of approximately 653.5 and 641.6 eV, respectively, indicating a mixed-valence manganese system [32]. The main peak of Mn 2p_3/2_ can be deconvoluted into two constituent peaks, corresponding to Mn^3+^ (641.5 eV) and Mn^4+^ (642.5 eV) [33]. The Mn 2p3/2 spectrum can only be deconvoluted into two peaks, whereas in other literature reports it has deconvoluted into three peaks, Mn^2+^ (640.7 eV), Mn^3+^ (641 eV), and Mn^4+^ (644 eV) [34,35]. This may be attributed to the presence of interactions between MnO_x_ and TiO_2_ through electron transfer. The valence state of manganese species is important for the redox properties of manganese-containing catalysts [36]. Figure 9b displays the fine spectrum of Ti 2p, whereas the Ti 2p energy spectrum shows a spin–orbit double consisting of Ti 2p_1/2_ and Ti 2p_3/2_ peaks with binding energies of about 463.28 eV and 457.78 eV, respectively. The main peak of Ti 2p_3/2_ can be resolved into two constituent peaks, corresponding to Ti^3+^ (458.48 eV) and Ti^4+^ (457.5 eV), respectively [36]. Similarly, the valence state of Ti species is of great significance for the redox properties of titanium-containing catalysts [29]. Figure 9c, conversely, presents the O 1s fine spectra of the samples, and the O 1s energy profile can be decomposed into three peaks corresponding to different types of oxygen on the catalyst surface. The peak at 529.5 eV is assigned to lattice oxygen (O latt, O^2−^), and the peak located at 531.1 eV is attributed to the surface adsorbed oxygen (O_2_ ads). The peak at 532.6 eV is associated with adsorbed molecular water and contaminated carbonate species (O_2_ other) [37], and it is generally believed that surface oxygen species are involved in oxidation reactions and that reactive surface oxygen promotes oxidation at low temperatures.

The proportions of the elements in each sample, in different valence states, are listed in Table 3. It can be observed that the relative percentages of Mn^4+^ are as follows: 700-12-3GN (32.45%) > 700-12-3LF (32.02%) > 700-12-3GN (30.36%); the relative percentages of Ti^4+^ are as follows: 700-12-3GN (47.34%) > 700-12-3LF (45.89%) > 700-12-3GN (40.09%); and the relative percentages of O_2_ ads are as follows: 700-12-3GN (27.87%) > 700-12-3LF (17.60%) > 700-12-3GN (16.93%). Studies in the literature have reported that the higher the content of Mn^4+^ and Ti^4+^ species in the catalyst, the stronger its redox performance as a catalyst [38,39]. This aligns with the XRD results.

**Figure 10:**
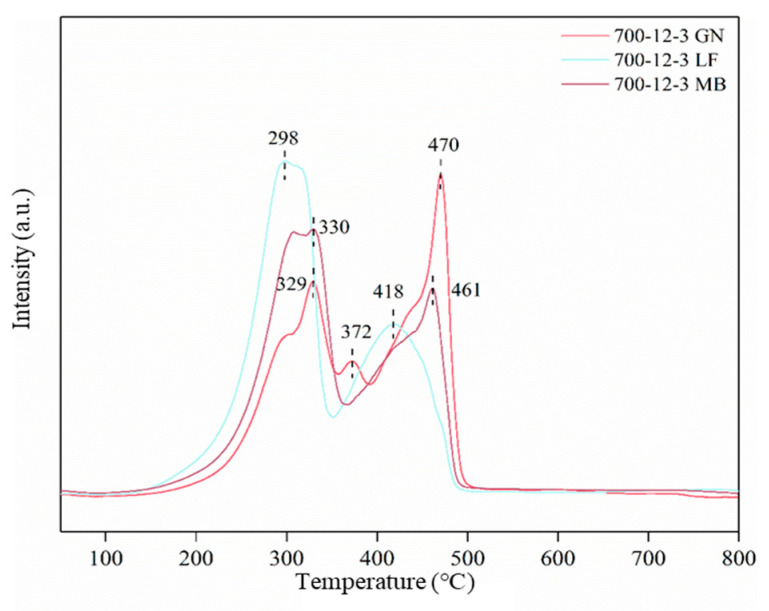
H_2_-TPR curve for catalysts.

Hydrogen temperature-programmed reduction (H_2_-TPR) enables the characterization of the redox properties of the catalyst surface. The H_2_-TPR profiles of the catalyst are depicted in Figure 10. As seen in Figure 10, 700-12-3GN exhibits three reduction peaks at the temperatures of 329 °C, 372 °C, and 470 °C; the other two samples exhibit only two reduction peaks: 700-12-3LF at 298 °C and 418 °C, and 700-12-3MB at 330 °C and 461 °C. The presence of the reduction peak suggests that the sample contains a significant amount of Mn^4+^ or Ti^4+^, leading to the formation of numerous reactive oxygen species crucial for the catalyst’s redox properties [36,40].

**Figure 11:**
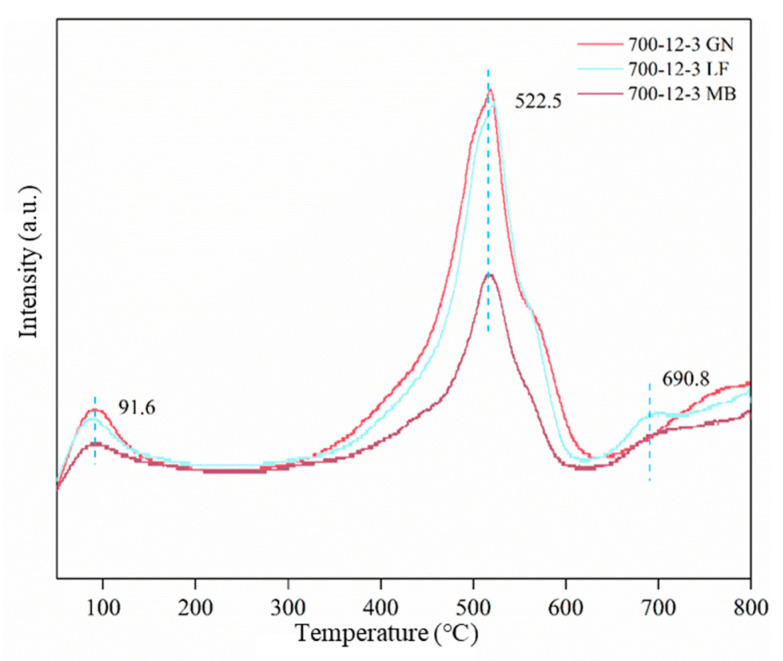
O_2_-TPD curve for catalysts.

Oxygen temperature-programmed desorption (O_2_-TPD) was used to investigate the nature of the oxygen species in the catalyst, and the O_2_-TPD profiles are presented in Figure 11. The O_2_-TPD curves of the catalysts reveal the oxygen storage capacity of the samples and their ability to utilize various oxygen species. The conversion path of adsorbed oxygen to lattice oxygen at the catalyst is predominantly governed by Equation [40]:

(3) $$O_{2}\left(\mathrm{ads}\right)\to O_{2}^{-}\left(\mathrm{ads}\right)\to O^{-}\left(\mathrm{ads}\right)\to O^{2-}\left(\mathrm{latt}\right)$$

Intensity peaks were observed in all three samples. Those within the temperature range of 60 to 260 °C can be attributed to the desorption of physically adsorbed oxygen molecules from the surface of the sample [41]; the intensity peaks between 260 °C and 600 °C are attributed to chemical adsorption on the surface of the catalysts and the surface-activated oxygen species. In contrast, the intensity peaks above 600 °C are attributed to the lattice oxygen of the metal oxides [42]. Among these, the peak intensity of 700-12-3GN was the largest, indicating that gaseous oxygen can be absorbed on the oxygen vacancies of the catalyst, forming adsorbed oxygen as an effective active species. Furthermore, photogenerated electrons can be trapped by oxygen vacancies, creating a trapping “well” that inhibits the recombination of photogenerated electrons with photogenerated holes, thereby enhancing the lifetime and quantum yield of photogenerated carriers [43].

**Figure 12:**
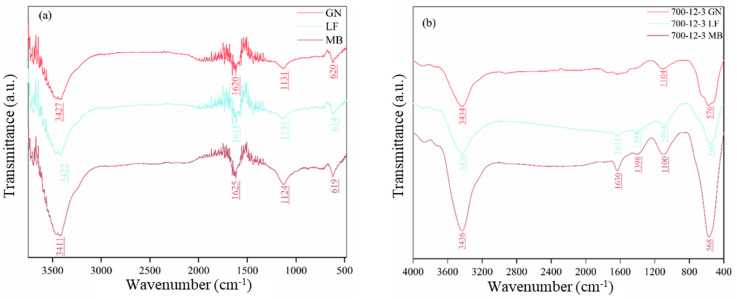
(**a**) FTIR map of the biochar, (**b**) FTIR map of the catalyst.

FTIR analysis was conducted to better understand the materials’ surface structure and potential reaction mechanisms. Figure 12a,b depict biochar without MnO_x_ and TiO_2_ and biochar loaded with MnO, and TiO_2_, respectively Among these, in Figure 12a, the peaks at 3427 cm^−1^, 3422 cm^−1^, and 3411 cm^−1^ correspond to intermolecular hydrogen bonding with the vibrational mode of -OH stretching [44]; the peaks at 1620 cm^−1^, 1611 cm^−1^, and 1625 cm^−1^ are associated with amides, exhibiting the vibrational mode of NH in-plane bending; and the peaks at 620 cm^−1^,614 cm^−1^, and 619 cm^−1^ are characteristic of alkynes, displaying the vibrational mode of CH out-of-plane bending [45]. These vibrational peaks indicate the presence of different functional groups on the surface of biochar. In Figure 12b, the peaks at 3434 cm^−1^, 3430 cm^−1^, and 3436 cm^−1^ correspond to intermolecular hydrogen bonding with the vibrational mode of -OH telescopic [46]; 1634 cm^−1^ and 1630 cm^−1^ represent groups belonging to olefinic hydrocarbons with the vibrational mode of C=C telescopic vibration [47]; and 1394 cm^−1^ and 1398 cm^−1^ represent groups belonging to olefins with the vibrational mode of CH- plane bending vibration [48]. Meanwhile, the peaks at 576 cm^−1^, 1094 cm^−1^, and 1100 cm^−1^ are characteristic of substituted benzenes, displaying the vibrational mode of CH- plane bending vibration [49], while 576 cm^−1^, 560 cm^−1^, and 568 cm^−1^ are the new peaks. Their representative groups belong to the MnO class, and the vibration mode is Mn-O stretching vibration [50], indicating the introduction of MnO. Based on the results, we hypothesized that the possible degradation mechanism of ethyl acetate is as follows [17]:

(4) $$TiO_{2}+hv\to e^{-}+h^{+}$$

(5) $$O_{2}\left(g\right)\to O_{2}\left(ads\right)$$

(6) $$4e^{-}+O_{2}\left(ads\right)\to 2O^{2-}$$

(7) $$O^{2-}+Mn^{4+}\to O\left(ads\right)+Mn^{2+}$$

(8) $$C_{4}H_{8}O_{2}\left(g\right)+e^{-}\to CH_{3}CH_{2}\cdot +CH_{3}COO\cdot$$

(9) $$CH_{3}COO\cdot +H\cdot \to CH_{3}COOH$$

(10) $$CH_{3}COOH+4O\cdot \to 2CO_{2}+2H_{2}O$$

(11) $$NO+CH_{3}COOH\cdot \to H_{2}O+CN$$

(12) $$2CN+O_{2}\left(\mathrm{ads}\right)\to 2NCO$$

(13) $$NCO+CH_{3}COOH\cdot \;\to N_{2}+CO_{2}+H_{2}O$$

## 4. Conclusions

Catalysts with varying loadings were prepared by sequentially loading MnO_x_ and TiO_2_ onto three types of biochar (prepared from GN, LF, and MB via a multi-step hydrothermal process). These catalysts were then utilized for the simultaneous removal of NO and ethyl acetate (EA) using the photothermal synergistic catalytic method. The catalysts were characterized and analyzed using XRD, BET, SEM, EDS, XPS, H_2_-TPR, and O_2_-TPD, and characterization methods were used to analyze the loaded catalysts. The results are summarized as follows: (1) Among the biochar pyrolyzed at 700 °C and the catalyst with a hydrothermal time of 12 h, the highest conversion efficiency of NO and EA was achieved at a catalyst dosage of 300 mg, with the 700-12-3GN catalyst exhibiting the most effective performance, reaching 73.66% and 62.09% conversions, respectively. (2) BET analysis revealed that different biochars led to variability in the specific surface area, pore volume, and average pore diameter of the samples. The specific surface area of 700-12-3GN was the smallest among the three samples, suggesting that a large specific surface alone does not determine the catalytic performance. (3) The successful loading of MnO_x_ and TiO_2_ on the biochar surface was demonstrated by FTIR, EDS, XPS, and XRD characterization analysis. It was found that different biochars led to variability in elemental content, and appropriate distribution and content of these elements are crucial for the conversion efficiency of NO. Among them, 700-12-3GN had the highest content of high-valence metal oxide species and surface oxygen species, contributing to the better catalytic activity of 700-12-3GN.

## Figures and Tables

**Table 1:** BET data for catalysts.

Sample	BET Surface Area (m^2^/g)	Pore Volume (cm^3^/g)	BJH Pore Diameter (nm)
700-12-3GN	53.65	0.254	17.3
700-12-3LF	72.87	0.233	11.2
700-12-3MB	61.38	0.249	14.5

**Table 2:** Atomic percentage of individual elements of the catalyst (%).

Element	700-12-3GN	700-12-3LF	700-12-3MB
Mn 2p	22.13	18.12	21.23
O 1s	51.4	47.54	50.34
Ti 2p	2.79	4.26	3.82

**Table 3:** Content of elemental valence in the prepared catalyst (%).

Catalyst	Mn^4+^/(Mn^4+^+ Mn^3+^)	Ti^4+^/(Ti^4+^+ Ti^3+^)	O_2_ ads/(O latt + O_2_ ads + O_2_ Other)
700-12-3GN	32.45	47.34	27.87
700-12-3LF	32.02	45.89	17.60
700-12-3MB	30.36	40.09	16.93

## Data Availability

Data are contained within the article and Supplementary Materials.

## Supplementary Materials

The following supporting information can be downloaded at: https://www.mdpi.com/article/10.3390/toxics12070478/s1, Figure S1: Experimental facility; Figure S2: Single thermal catalytic activity of 700-12-3GN catalyst; Figure S3: XPS full spectrum of the catalyst.
