# Supplementary material for: Optimization of Photothermal Catalytic Reaction of Ethyl Acetate and NO Catalyzed by Biochar-Supported MnOx-TiO2 Catalysts
Source: Toxics. 2024 Jun 30;12(7):478. doi: 10.3390/toxics12070478 (PMC11280807; doi:10.3390/toxics12070478)
Supplement: Supplementary file 1 [file toxics-12-00478-s001.zip › toxics-3034399-supplementary.pdf]

Optimization of Photothermal Cocatalytic Reaction of Ethyl Acetate and NO catalyzed by Biochar-supported MnO_x-TiO_2 Catalysts

Hongqiang Wang[1], Huan Zhang[1], Luye Wang[1], Lei Liao[1], Shengpeng Mo[1], Xiaobin Zhou[1], Yanping Zhang[1], Yinming Fan[1,2*]

[1] School of Environmental Science and Engineering, Guilin University of Technology, Guilin 541000, China

[2] The Guangxi Key Laboratory of Theory and Technology for Environmental Pollution Control, Guilin University of Technology, Guilin 541000, China

[3] Guangxi Collaborative Innovation Center for Water Pollution Control and Water Safety in Karst Areas, Guilin 541004, China

1. Catalyst preparation

1.1 Synthesis of biochar

Three kinds of biomass raw materials (ginkgo shell, loofah, and moso bamboo) were washed and the surface moisture was dried at 80°C. The pyrolysis was carried out in a muffle furnace at a heating rate of 1°C/min and heated to 700°C for 2 h. After natural cooling to room temperature, the samples were taken out, washed with ultra-pure water, dried at 80°C, and screened to obtain 100 mesh samples, named GN, LF, and MB.

1.2 Preparation of biochar catalyst

For the preparation of the biochar catalyst, 3.4 g of manganese sulfate monohydrate ($MnSO_4 \cdot 4H_2O$) was dissolved in 30 ml of ultrapure water, named A, and 16.9 g of ammonium persulfate [$(NH_4)_2S_2O_8$] was dissolved in 30 ml of ultrapure water, named B. After stirring at room temperature for 15 min, A was added to B, stirring was continued for 15 min, and ultrasonically dispersed for 15 min. Then, 500 mg of 300 mg of 100 mg of biochar was dispersed into the mixed solution, stirred for 10 min, and sonicated for 20 min, respectively. The dispersed solution was then transferred to a reactor and held at 80°C for 4 h. After cooling to room temperature, the mixture was washed with ultrapure water until the filtrate was clear and dried at 80°C for 12 h to obtain the mixture.

After, 6.67 mL of tetrabutyl titanate was mixed with 60 mL of anhydrous ethanol, stirred for 15 min, dispersed by ultrasonication for 15 min, added to the above mixture, stirred for 10 min, dispersed by ultrasonication for 20 min, and transferred to a reactor for 12 h at 80°C with hydrothermal heat. After the reaction, the mixture was washed several times with anhydrous ethanol and dried at 80°C. The resulting mixture was heated to 700°C in a tube furnace at an elevated rate of 2°C/min and held for 2 h. Finally, the obtained catalysts were pressed under 10 MPa pressure to take 40 mesh to 60 mesh and named as 700-12-5GN, 700-12-5LF, 700-12-5MB, 700-12-3GN, 700-12-3LF, 700-12-3MB, 700-12-1GN, 700-12-1LF, 700-12-1MB.

2. Catalysts characterization

The crystalline structure of the catalysts was analyzed by X-ray powder diffraction (XRD, X′Pert3 Power). The phototube was a Cu-target ceramic phototube with a tube voltage of 40 kV, a tube current of 40 mA (λ=1.5406 Å), and X-ray scanning time, swept range, and scanning step size of 15 min, 5~90°, and 0.02, respectively.

The specific surface area (Brunauer–Emmett–Teller model) and pore size distribution (Barrett–Joyner–Halenda model) of each catalyst were measured using Micromeritics ASAP 2020. The catalysts were degassed at 250°C for 8 h prior to measurement to minimize the effect of physiosorbed impurities.

The surface morphology of the catalysts was characterized by scanning electron microscopy (SEM, TESCAN MIRA LMS) with an energy dispersive X-ray attachment (EDS).

X-ray photoelectron spectroscopy (XPS) studies were carried out a monochromatic Al Ka (hv=1486.6 eV) radiation was carried out by Thermo Scientific K-Alpha electron spectrometer. The binding energies were calibrated based on the C 1s peak at 284.7 eV, and the surface compositions and chemical states of Ti 2p, Mn 2p, and O 1s were evaluated by calculating the areas of the photoelectron peaks and the binding energies.

The catalysts were characterized for H_2 temperature-programmed reduction (H_2-TPR) and O_2 temperature-programmed desorption (O_2-TPD) using Autochem II 2920 Micromeritics. When testing H_2-TPR, 30 mg of catalyst was weighed and placed in a quartz reactor, preheated from room temperature to 200°C, and kept in an Ar gas stream for 1 h (30 mL·min^{-1}). After cooling to 50°C, the catalyst was heated from room temperature to 600°C in 10% H_2/Ar (30 mL·min^{-1}) at a rate of 10 °C·min^{-1}. When testing the O_2-TPD, 30 mg of catalyst was weighed and placed in a 30 mL·min^{-1} O_2 gas stream and held at 250°C for 1 h. After cooling to 50°C, the catalyst was heated from room temperature to 800°C in a pure He gas stream (30 mL·min^{-1}) at a rate of 10°C·min^{-1}.

The chemical composition of the catalysts was analyzed by Fourier infrared spectroscopy (FT-IR, Frontier).

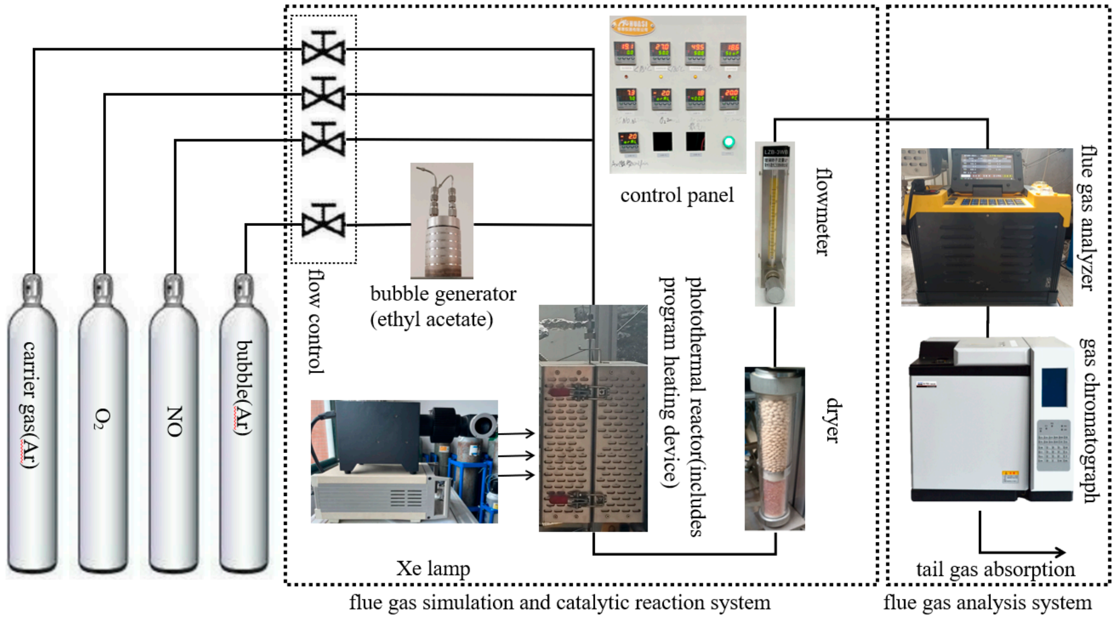

Figure S1 Experimental facility.

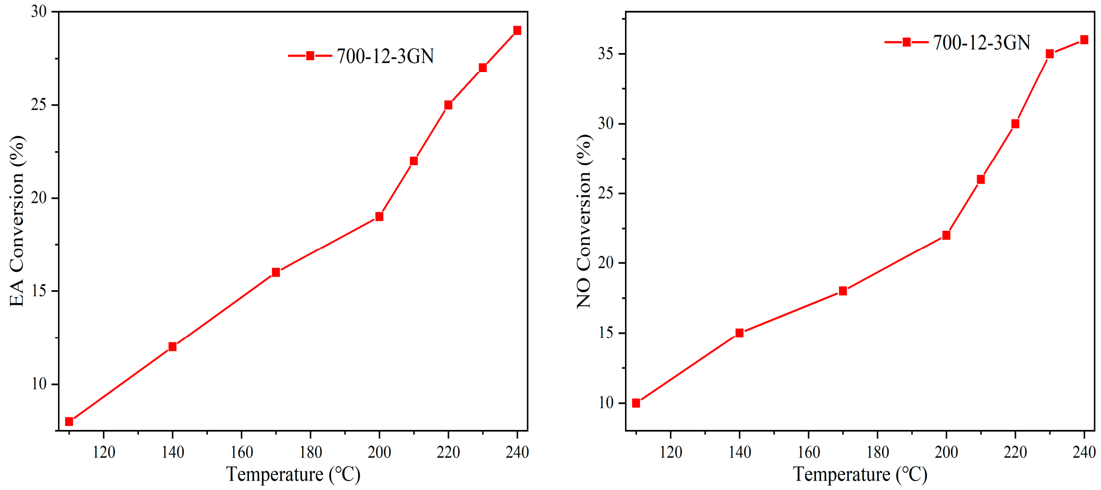

Figure S2. Single thermal catalytic activity of 700-12-3GN catalyst.

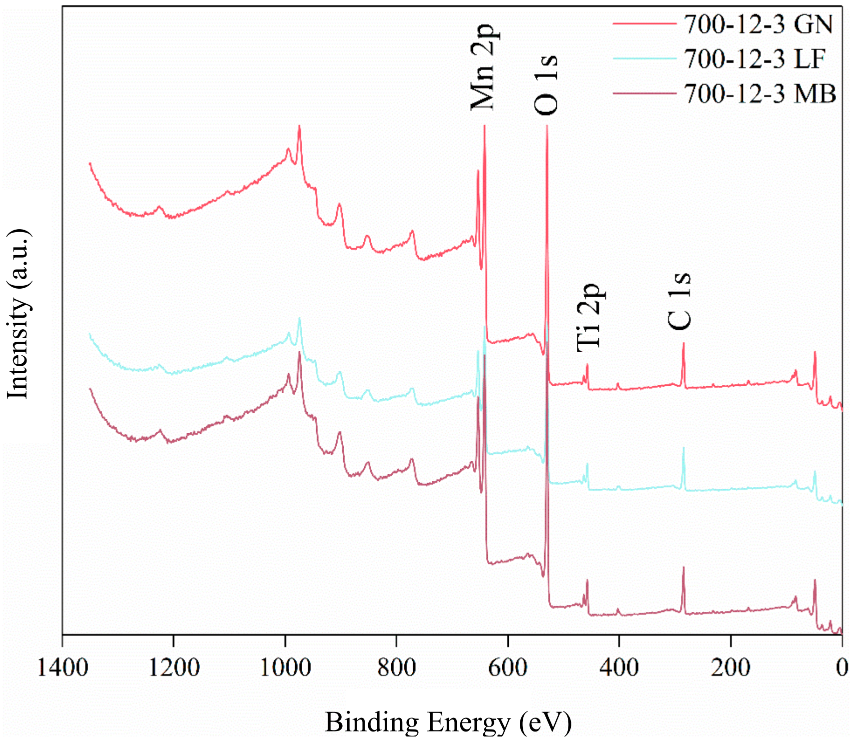

Figure S3. XPS full spectrum of the catalyst.
